# Antioxidant and Antisteatotic Activities of a New Fucoidan Extracted from *Ferula hermonis* Roots Harvested on Lebanese Mountains

**DOI:** 10.3390/molecules26041161

**Published:** 2021-02-22

**Authors:** Zeinab El Rashed, Giulio Lupidi, Hussein Kanaan, Elena Grasselli, Laura Canesi, Hala Khalifeh, Ilaria Demori

**Affiliations:** 1 Department of Earth, Environmental and Life Sciences (DISTAV), University of Genoa, 16132 Genoa, Italy; zeinab.alrashed94@hotmail.com (Z.E.R.); elena.grasselli@unige.it (E.G.); laura.canesi@unige.it (L.C.); 2 Rammal Rammal Laboratory (ATAC Group), Faculty of Sciences I, Lebanese University, 1003 Beirut, Lebanon; hala-khalifeh@hotmail.com; 3 School of Pharmacy, University of Camerino, 62032 Camerino, Italy; giulio.lupidi@unicam.it; 4 Laboratory of Chemical Synthesis and Extraction of Polysaccharides from Seaweed, Faculty of Pharmacy, Lebanese University, 1003 Beirut, Lebanon; hkanaan@ul.edu.lb

**Keywords:** *Ferula hermonis*, fucoidan, antioxidant, antisteatotic, NAFLD

## Abstract

Fucoidan is a fucose-rich sulfated polysaccharide with attractive therapeutic potential due to a variety of biological activities, including antioxidant action. Fucoidan is typically found in the cell wall of marine brown algae, but extra-algal sources have also been discovered. In the present work, for the first time we extracted a water soluble fucoidan fraction from the roots of the terrestrial shrub *Ferula hermonis.* This fucoidan fraction was termed FUFe, and contained fucose, glucose, sulfate, smaller amounts of monosaccharides such as galactose and mannose, and a minor quantity of proteins. FUFe structural features were investigated by FTIR, ^1^H NMR and ^13^C NMR spectroscopy. The antioxidant property of FUFe was measured by DPPH, ABTS and FRAP assays, which revealed a high radical scavenging capacity that was confirmed in in vitro cellular models. In hepatic and endothelial cells, 50 μg/mL FUFe could reduce ROS production induced by intracellular lipid accumulation. Moreover, in hepatic cells FUFe exhibited a significant antisteatotic action, being able to reduce intracellular triglyceride content and to regulate the expression of key genes of hepatic lipid metabolism. Altogether, our results candidate FUFe as a possible bioactive compound against fatty liver disease and related vascular damage.

## 1. Introduction

Since ancient times, plants have been used as a medical source for treating human diseases [1]. Nowadays, the search for natural compounds displaying therapeutic efficacy with less toxicity and side effects than synthetic drugs is still of great interest. Many phytochemicals are currently being studied for their potential benefits against the major threats to human health, i.e., non-communicable diseases including cardiovascular disorders, cancer, autoimmunity, neurodegenerative diseases, as well as metabolic impairments related to insulin resistance and liver disorders, such as nonalcoholic fatty liver disease (NAFLD). Oxidative stress and reactive oxygen species (ROS) overproduction are considered as common denominators of such a variety of pathologies; hence, in a preventive and therapeutic perspective, many efforts are being made to discover and characterize the antioxidant properties of natural compounds [2].

Fucoidan (FU) is an example of phytochemical that gained a great interest in research due to its antioxidant and anti-inflammatory activities [3,4]. FU is a fucose-rich sulfated polysaccharide with a backbone of α-(1–3)-linked fucose units or α-(1–3)- and α-(1–4)- alternating linked fucose residues. It is abundant in the cell wall of marine brown algae, but also found in marine invertebrates, such as in the jelly coat from sea urchin eggs, and in the sea cucumber body wall [5]. Recently, it has been identified also in the terrestrial tree *Eucaliptus globulus* [6], which is widely known for different uses in herbal medicine. This latter finding inspired us to investigate the presence and properties of FU from another medicinal terrestrial plant, i.e., *Ferula hermonis,* a small shrub belonging to the Apiaceae family. *F. hermonis* is endemic in Lebanon, Syria and Jordan, where the roots, harvested from August to October, are traditionally used to enhance sexual behavior and treat infertility and menopausal disturbances, so that it is commonly known as “Shirsh El Zallouh” or “Lebanese Viagra” [7]. Moreover, it has been shown that *F. hermonis* exerts antibacterial, anti-inflammatory, antidiabetic, hypolipidemic, and hepatoprotective effects. These biological activities are related to the presence of a wide range of active compounds such as sulfur-containing compounds, sesquiterpenes, coumarins, ferutinin, α-pinene, camphene and carvacrol, as well as vitamins (A and E) and some minerals [7]. According to the chemical composition reported by Abdel-Kader et al. [8], *F. hermonis* roots contain 38.38% carbohydrates, 8.33% lipids, and 3.48% proteins; however, to our knowledge, no investigation has been conducted on the water-soluble polysaccharide fractions. In the present work, for the first time we extracted and structurally characterized a FU fraction from *F. hermonis* roots. We termed this extract FUFe and we evaluated its radical scavenging capacity, as well as its antioxidant and antisteatotic effects using in vitro cellular models of NAFLD and endothelial damage.

## 2. Results

### 2.1. Extraction Yield and Chemical Composition

The yield of the FU extraction from *F. hermonis* roots was 3.07%. The chemical composition of the extract is shown in Table 1. The analyses confirmed that FUFe was composed primarily of fucose with an amount of sulfate groups of 1.8% (molar ratio respect to fucose 1:0.1). The content of glucose was the highest respect to other monosaccharides such as galactose and mannose. The molar ratio in native polysaccharide was estimated to be about 1.1:1.0:0.1:0.05 for fucose, glucose, galactose, and mannose, respectively. The low protein content indicated a high degree of purity of the FUFe extract.

### 2.2. Spectroscopic Characterization

The FUFe extract was subjected to preliminary structural analysis (spectra are reported in Supplementary Materials). As shown in Figure S1, Supplementary Materials, the FTIR spectrum of FUFe showed a wide band centered at 3301 cm^−1^, which was assigned to hydrogen bonded O–H stretching vibration. The weak signal at 2926 cm^−1^ indicated the presence of C–H [9]. The band at 1595 cm^−1^ indicated the absorbance of uronic acid carbonyl group C=O, while that at 1410 cm^−1^ was assigned to carboxylate group vibrations (C–O). The weak band at 1273 cm^−1^ indicated the presence of S=O stretching vibration of the sulfate group, and the absorption at 1028 cm^−1^ was due to a sulfate ester group [10]. Additional band at 531 cm^−1^ indicated the presence of C–O–S sulfate residues [11].

The Proton (^1^H) NMR analysis of FUFe is shown in Figure S2. The ^1^H NMR spectrum contained chemical shifts ranging from 5.0 to 5.7 ppm that are consistent with the presence of protons (H1α) of α-linked-l-fucose and β-linked sugars (H1β). Chemical shifts indicated between 0.75 and 1.81 ppm corresponded to the methyl group which is a main characterization of L-fucopyranose (L-FucP). The spectrum contained resonance characteristics of FU with signals from ring protons (H-2 to H-5) at 3.46–4.20 ppm that support the presence of different types of fucosal sulfate groups with changes in glycosidic linkage positions and monosaccharide patterns [12,13]. Furthermore, ^1^H signals due to linear unsubstituted L-fucose resonated at 5.07 to 5.22 ppm and the signals at 5.38–5.39 ppm could be supposed signals of substituted L-fucose and side chain of D-pyranose residues [14].

The ^13^C NMR spectrum of FUFe reported in Figure S3 showed major signals of sulfated L-fucan between 92.2 and 103.7 ppm (C1) and at 16.6 ppm (C6). The signals obtained in the region 60.2 and 82.0 ppm corresponded to pyranoid ring carbons (C2–C5) [12,15].

In the ^13^C NMR spectrum of FUFe there are at least seven separate anomeric carbon signals. Analysis of monosaccharide components reported in Table 1 indicated the presence of fucose and glucose in similar molar ratio (1.1:1) and traces of galactose and mannose, so the degree of heterogeneity is high. The C6 signal of presumably glucose is visible at 62.4 ppm, but some signals, for example those at 61.4 and 60.1.3 ppm, probably come neither from fucose nor from other monosaccharides, being rather due to unidentified impurities.

### 2.3. Radical Scavenging Capacity

DPPH, ABTS and FRAP assays were used as easy, rapid and sensitive methods to determine the scavenging capacity of FUFe against different radicals. The results are summarized in Table 2. As indicated, FUFe showed a good ability in scavenging the DPPH free radical (about 20% to 76% inhibition in the concentration range 50–500 µg/mL), resulting in an antioxidant activity about 38 times lower than ascorbic acid (IC_50_ for ascorbic acid was 4.14 ± 2.12 µg/mL).

Table 3 shows the results of ABTS and FRAP assays. A high scavenging capacity was measured by ABTS assay, in which FUFe antioxidant activity was about 10 times less than Trolox. The FRAP value reflected the significant antioxidant activity of FUFe, that could be attributed to different mechanisms, such as complex with transition metal ion catalysts, break of chain initiation, radical scavenging prevention and increase of reductive capacity.

Similar assays have been performed with other water-soluble polysaccharides and fucoidan fractions previously characterized from different sources [4,6,13,14,16,17]. Table 4 shows a comparison of maximum RSA for FUFe with respect to other terrestrial and marine sources of FU extracted following the same procedures [6,14]. FUFe displays a higher radical scavenging potential with respect to FU from the brown algae *Stypopodium schimperi*, but it is a weaker antioxidant when compared to FU recently identified in another Lebanese terrestrial plant, *Eucalyptus globulus.*

### 2.4. Antioxidant and Antisteatotic Activities of FUFe in a Cellular Model of Hepatic Steatosis

We used an in vitro model of hepatic steatosis consisting in FaO cells overloaded with a mixture of oleate/palmitate (OP, 0.75 mM) to mimic nonalcoholic fatty liver disease (NAFLD) [18]. Preliminary experiments demonstrated that FUFe at different concentrations (5–100 μg/mL) did not affect FaO cell viability both in the absence and in the presence of OP, as measured by MTT assay (data not shown). The intermediate dose of 50 μg/mL FUFe was selected for further experiments.

**Figure 1:**
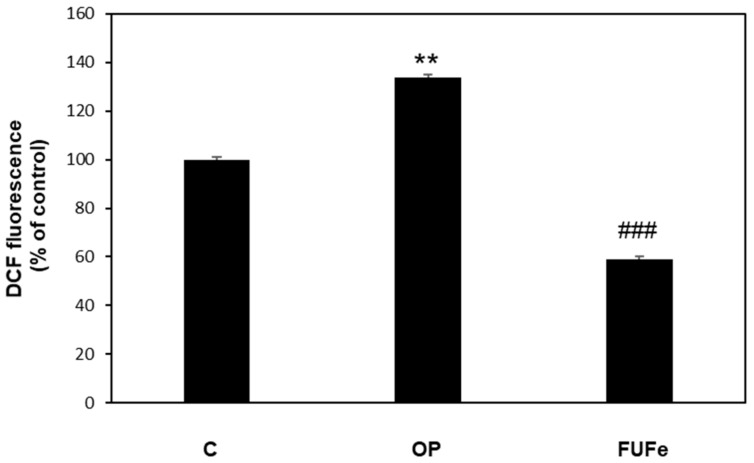
Effects of FUFe on ROS production in steatotic FaO cells DCF fluorescence was quantified in control (C) and steatotic cells incubated in the absence (OP) or in the presence of 50 μg/mL FUFe for 24 h. Data are expressed as percentage of control. Values are mean ± S.D. from three independent experiments. Significant differences are denoted by symbols: ** *p* ≤ 0.01 vs. control; ### *p* ≤ 0.001 vs. OP.

The antioxidant ability of FUFe was analyzed using 2′,7′-dichlorofluorescein diacetate (DCF-DA) assay for ROS detection in FaO cells (Figure 1). OP treatment resulted in a significant increase in ROS production (+30%, *p* ≤ 0.01 with respect to control), but FUFe was able to significantly reduce ROS production by more than 50% as compared to steatotic cells (*p* ≤ 0.001 vs. OP).

**Figure 2:**
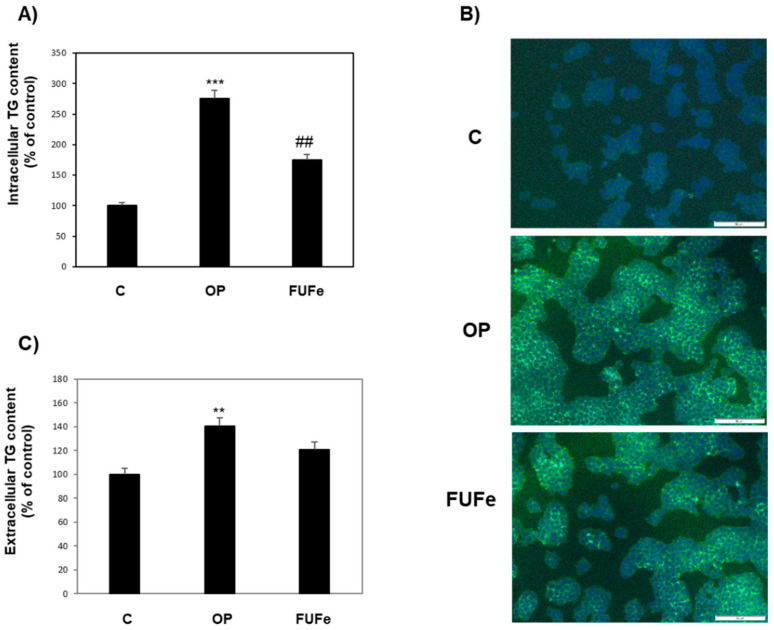
Antisteatotic effect of FUFe on FaO cells. (**A**) Intracellular TG content in control (C) and steatotic cells incubated in the absence (OP) or in the presence of 50 μg/mL FUFe for 24 h. (**B**) Representative images of BODIPY (green)/DAPI (blue) staining of FaO cells showing cytosolic LDs (Magnification 10×; bar 50 μm). (**C**) Extracellular TG content as measured in the culture medium of the same cells. Data are expressed as percentage of control. Values are mean ± S.D. from three independent experiments. Significant differences are denoted by symbols: ** *p* ≤ 0.01, *** *p* ≤ 0.001 vs. C; ## *p* ≤ 0.01 vs. OP.

The antisteatotic action of FUFe was assessed by measuring triglyceride (TG) accumulation in FaO cells (Figure 2). As previously described [19], OP treatment resulted in a significant increase in TG content with respect to control (+175%; *p* ≤ 0.001). However, incubation with 50 μg/mL FUFe after OP treatment decreased TG content by 36% with respect to OP (*p* ≤ 0.01) (Figure 2A). These results were confirmed by fluorescence microscopy visualization of intracellular lipid droplets (LDs) stained in green with BODIPY 493/503 (Figure 2B). We also measured extracellular TG content in cell culture medium as an indication of TG secretion by FaO cells. Figure 2C shows that TG secretion was significantly increased in OP cells with respect to control (+40%, *p* ≤ 0.01), and that subsequent FUFe treatment did not affect this value.

**Figure 3:**
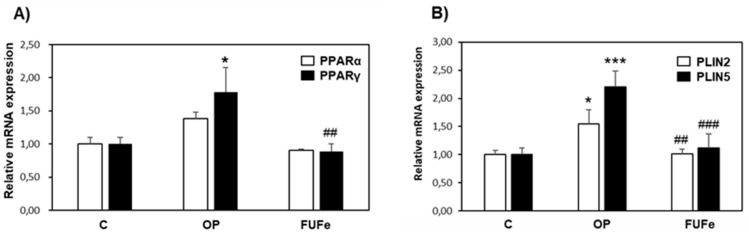
Effects of FUFe on hepatic gene expression. The relative mRNA expression of (**A**) PPARα and PPARγ and (**B**) PLIN2 and PLIN5 was quantified by qPCR in control (C) and steatotic FaO cells incubated in the absence (OP) or in the presence (FUFe) of 50 µg/mL FUFe for 24 h. Data are expressed as fold induction with respect to controls. Values are mean ± S.D. from three independent experiments. Significant differences are denoted by symbols: * *p* ≤ 0.05, *** *p* ≤ 0.001 vs. C; ## *p* ≤ 0.01, ### *p* ≤ 0.001 vs. OP.

To further explore the antisteatotic effects of FUFe, we used qPCR to investigate the expression of key genes of hepatic lipid metabolism, such as those coding for PPAR (peroxisome proliferator-activated receptor) transcription factors [20] and for a family of LD-associated proteins (perilipins, PLINs) [21]. The most abundant PPAR isoforms expressed in FaO cells are PPARα and PPARγ [19] and Figure 3A shows the results of cell treatments on their expressions. For PPARα, a slight increase in steatotic cells and a return to control values after FUFe treatment was apparent, but the results did not reach statistical significance. On the contrary, PPARγ expression was significantly induced by OP treatment (1.5-fold induction, *p* ≤ 0.05) and a significant down-regulation to control levels was measured after incubation with FUFe (*p* ≤ 0.01 as compared to OP). PLIN2 and PLIN5 hepatic expressions are regulated by PPARγ [22,23]. Accordingly, the mRNA levels of both PLINs are significantly increased in steatotic FaO cells with respect to control (1.5- and 2- fold induction, *p* ≤ 0.05 and *p* ≤ 0.001, respectively) and down-regulated to control values by FUFe incubation (*p* ≤ 0.01 and *p* ≤ 0.001 for PLIN2 and PLIN5, respectively; Figure 3B).

### 2.5. Effects of FUFe on Steatotic Endothelial Cells

**Figure 4:**
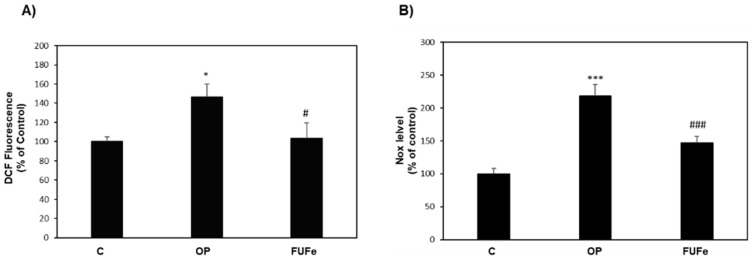
Effects of FUFe in steatotic HECV cells. (**A**) DCF fluorescence and (**B**) NOx production were quantified in control (C) and steatotic cells incubated in the absence (OP) or in the presence of 50 μg/mL FUFe for 24 h. Data are expressed as percentage relative to control. Values are mean ± S.D. from three independent experiments. Significant differences are denoted by symbols: * *p* ≤ 0.05, *** *p* ≤ 0.001 vs. C; # *p* ≤ 0.05, ### *p* ≤ 0.001 vs. OP.

OP-treated HECV cells can be considered as a model of atherosclerosis and endothelial damage [24]. As for FaO cells, OP treatment of HECV cells resulted in a significant increase in ROS production (+46%, *p* ≤ 0.05 with respect to control), as measured by DCF assay. In OP cells, FUFe was able to significantly reduce ROS production to control levels (*p* ≤ 0.05 vs. OP) (Figure 4A). As shown in Figure 4B, OP treatment also significantly enhanced NO production in HECV cells compared to control (+118%, *p* ≤ 0.001) and this increase was effectively reduced by FUFe treatment (−33%, *p* ≤ 0.001 with respect to OP).

## 3. Discussion

Fucoidan (FU) is a water-soluble polysaccharide that possesses a variety of potential therapeutic and pharmaceutic applications, due to a wide spectrum of biological activities [3,4]. The main source of FU is the marine environment, particularly brown algae, such as *Fucus vesiculosus* [5]. To our knowledge, this is the first time that a FU has been isolated and characterized from the roots of *F. hermonis,* a terrestrial shrub endemic in Lebanon, where it is known and currently used as a medicinal plant. Of note, the extraction yield of FU from *F. hermonis* (FUFe) (3.07%) was higher than that recently obtained from another terrestrial plant, i.e., *E. globulus* growing in Lebanon (2.1%) [6]. On the other hand, FUFe yield is smaller when compared to those reported for marine sources: for example, FU extraction yield from the brown seaweed *Cystoseira barbata* is 6.44% [25].

The chemical composition of FUs extracted from different sources can be relatively complex, with traces of different sugars which increase the difficulty of structural analysis [5]. The analysis of FUFe composition revealed the presence of minor monosaccharides (galactose and mannose), considerable amounts of fucose and a comparable content of glucose. The latter seems to be a peculiarity of FUFe structure, which deserves further investigations. The low protein contamination of FUFe (about 0.5%) indicated a high level of purity as compared with similar extracts. In fact, a protein content ranging from 0.2% to 2.5% has been reported in FUs extracted from different sources [26,27,28]. We tried to further purify FUFe with advanced methods such as gel filtration, but it reduced the FU yield and did not change protein content significantly. It is also well known that several factors including extraction method, vegetal species, seasonal variations, and reproductive status of the plants can affect the yield and chemical composition of purified FUs [29].

A preliminary structural analysis of FUFe was performed by FTIR and NMR spectroscopy (spectra are reported in Supplementary Materials), which confirmed the presence of functional groups, such as fucopyranosyl units and sulfate groups, that characterize FU. Some variations in the peaks that can be appreciated when comparing data obtained from *F. hermonis* to those from *E. globulus* or *Dictyopteris polypodioides* [6,15] could be related to the different species and the diversity of the growth environment. Together with differences in chemical composition, these variations likely affect the bioactivity of purified FU [30].

Based on our data, we can hypothesize for FUFe a linear chain of alternating (1,3)- or (1,6)-linked α-L- fucopyranose residues (103.6, 92.2 ppm) with sulfate groups at positions 2 (82.0 ppm). However, further spectrophotometric techniques and chemical characterization studies are currently planned to better investigate the detailed structure of FUFe regarding sulfation, O-acetylation, linkage, and branching points of the backbone chain. This will help to understand the relationships between the chemical structure of FUFe and its biological activities that remain to be established.

Being oxidative stress a common cause of most of the non-communicable diseases of the modern age, the antioxidant activity is perhaps one of the main biological potentialities of phytochemicals in general and of FU in particular. The hydroxyl hydrogen donor groups detected by FTIR spectroscopy could contribute to the antioxidant potential of FUFe. We used three different assays to characterize the antioxidant capacity of FUFe, and our data showed that it exhibited a high scavenging activity against DPPH and ABTS radicals, as well as a ferric reducing power. Compounds or extracts displaying IC_50_ values ranging from 50 to 100 μg/mL in different antioxidant assays are considered as strong antioxidants [31]. As reported in Table 2 and Table 3, FUFe IC_50_ was 38 times lower than ascorbic acid in DPPH assay and 10 times less than Trolox in ABTS assay. Table 4 shows that at the concentration of 500 μg/mL, FUFe as an antioxidant was weaker than FU extract from *E. globulus*, but stronger than FU fraction from *Stypopodium schimperi* [6,14]. This confirms that the radical scavenging activity of FUs depends on vegetal species as well as on environmental conditions. Other reports [13,16] showed high antioxidant abilities in several FUs from brown algae (*Sargassum filipendula, Cladosiphon okamuranus, Sargassum hornery, Kjellmaniella crassifolia, Nemacystus decipiens*, and *Fucus vesiculosus*), with different strength depending on the species (IC_50_ ranging from 1.15 to 4.50 mg Trolox/g FU), and suggest that a combination of factors, such as the amount and position of sulfate groups, the kind of side chain sugar, and the molecular weight, all contribute to determine the final antioxidant action of different FU extracts [13].

Besides in vitro antioxidant assays, it is of note that radical scavenging capacity of FUFe was confirmed in cellular systems, pointing to beneficial actions of FUFe as a phytochemical.

First, we focused on cellular models related to NAFLD and endothelial damage. NAFLD is a physio-pathological condition affecting 25% of the population worldwide; it does not simply represent a fatty liver, because its worsening is a burden to the liver and extrahepatic tissues as well, being closely associated with cardiovascular diseases [32]. Apart from dietary and lifestyle interventions, there is still a lack of therapies for NAFLD, and research is addressed to discover new natural compounds able to counteract NAFLD onset and progression, possibly without the side effects of synthetic drugs. The literature about algae as nutritional and functional food sources, is extremely rich [33] and FUs from different seaweeds are the most extensively studied algal polysaccharides in relation to NAFLD, showing efficacy both in in vivo and in vitro experimental models, as we recently reviewed [34].

In our NAFLD model of OP-treated FaO cells [18], 50 μg/mL FUFe down-regulated ROS production induced by excessive fat. Oxidative stress can be considered as the starting point of the hepatic damage, contributing to immunological dysfunction and inflammation, thus triggering NAFLD progression and worsening toward more severe hepatic damage [35]. Accordingly, the antioxidant effect of FUFe was associated with an antisteatotic action, resulting in a significant decrease in the intracellular TG content in lipid-overloaded FaO cells.

To further investigate the antisteatotic effect of FUFe in FaO cells, we measured the expression level of peroxisome proliferator-activated receptors (PPARs). PPARs are ligand-activated transcription factors that play a key role in the regulation of hepatic lipid balance, and their expression is regulated in NAFLD and by NAFLD therapies [20]. As previously reported, in lipid-overloaded FaO cells hepatic steatosis was associated with an increase in PPARγ expression [18]. PPARγ is a known marker of fatty liver and its expression is induced to allow energy storage, as it mediates the activation of lipogenic genes [36,37]. Accordingly, the decrease in intracellular fat content elicited by FUFe treatment of steatotic FaO cells was associated with a down-regulation of PPARγ expression toward control levels. In the liver, PPARγ appears to regulate the expression of PLIN2 and PLIN5 [22,23]. PLINs (perilipins) are a family of LD-associated proteins that confer dynamicity to these organelles and play important roles in their metabolism, taking part in the regulation of lipid trafficking inside and outside the cell [21,38]. In our NAFLD model, PLIN2 and PLIN5 displayed the same pattern of expression of PPARγ, being significantly enhanced in steatotic cells and decreased after FUFe treatment. Taken together, these results confirm the antisteatotic action of FUFe demonstrating a down-regulation of energy storing mechanisms with the involvement of changes in LD trafficking, which are also documented by the representative image of BODIPY staining for neutral lipids, showing less LDs in FUFe-treated steatotic FaO cells. Excess fats mobilized from LDs could be addressed to secretion or oxidative catabolic pathways. It was beyond the scope of this paper to further investigate these mechanisms; however, the lack of effect of FUFe on TAG released by steatotic cells in the culture medium might indicate that lipid secretion is not directly stimulated by FUFe.

In NAFLD, endothelial cells of the hepatic sinusoids participate in the development toward liver inflammation and fibrosis [39] and endothelial functions are impaired in metabolic disorders since the endothelium represents the interface between blood and tissues to allow energy supply [40]. Therefore, we investigated the effects of FUFe also in human endothelial cells (HECV). Previous data showed that these cells cultured in the presence of fatty acids accumulate cytosolic lipids and could be considered as a model of atherosclerosis [24]. In HECV cells, ROS production was enhanced by OP treatment, but decreased to control values after FUFe incubation, thus confirming the antioxidant capacity of FUFe in cellular systems. Moreover, in our model lipid overload and oxidative stress were associated with an increase in NO production, which was lowered by FUFe treatment. It is well known that in liver sinusoids, NO influences endothelial cell functions related to inflammatory and fibrotic processes [39]. Moreover, NO regulates vascular tone and blood flow by activating soluble guanylate cyclase in the vascular smooth muscle, and it is essential for leukocyte adhesion and platelet aggregation. As such, our results suggest the potentiality of FUFe to counteract endothelial dysfunction in cardiovascular pathologies such as hypertension and atherosclerosis, which are often associated with NAFLD [41].

## 4. Materials and Methods

### 4.1. Biological Material and Chemicals

Roots of *F. hermonis* were harvested from “Mountains of Bekaa”, Lebanon, in August 2017. The plant was authenticated by Dr. George Tohme, Professor of Taxonomy and President of the National Council for Scientific Research (CNRS), Beirut, Lebanon. A voucher specimen (PS-14-16) has been deposited in the Herbarium of the Faculty of Science, Lebanese University, Hadath, Beirut, Lebanon. Harvested roots were washed and cleaned with water, then dried at room temperature and grinded for further extraction. Unless otherwise indicated, all chemicals were supplied by Sigma-Aldrich and of analytical or cell culture grade.

### 4.2. Extraction and Purification of Fucoidan

Water-soluble polysaccharides were obtained as previously described [6] using slightly modified methods [42,43]. Briefly, 100 g of *F. hermonis* roots were extracted twice with 250 mL absolute ethanol for 3 h at 40 °C to remove low-molecular-weight compounds such as pigments, phenols, and proteins. The dried residues were extracted twice with aqueous HCl solution (pH = 2) at 60 °C for 3 h, then centrifuged at 1600× *g* for 20 min to obtain supernatant, containing the FLM complex (Fucoidan, Laminarin, Mannuronan). The supernatant was neutralized with 3% NaHCO_3_, evaporated in a Rotavac Vario Power Unit (Heidolph Instruments, Schwabach, Germany) to a final volume of 200 mL, which proceeded to 24 h dialysis (Spectra/Por Dialysis Tubing, MWCO 12000-14000) and subsequent lyophilization to obtain FLM powder. Fucoidan purification was obtained by adding 50 mL of aqueous HCl solution (pH = 2), followed by centrifugation at 1600× *g* for 20 min. The pellet was discarded whereas supernatant was lyophilized to obtain dry powdered fucoidan (FUFe), which was weighed to calculate yield.

### 4.3. Chemical Characterization

Sulfate and protein contents were determined by the turbidimetric assay described by Jackson and McCandless [44]. Absorbance was read at 500 nm with a Varian Cary 50 UV-VIS spectrophotometer (Agilent, Milan, Italy). Sugar content was quantified using the phenol-sulfuric acid method developed by Dubois et al. [39]. Absorbance was recorded at 520 nm. Fourier Transform Infrared spectroscopy (FTIR) of FUFe was recorded on a Perkin-Elmer FTIR spectrometer Spectrum Two UAT. Data were collected in the range of 4000–400 cm^−1^. Proton (^1^H NMR) and carbon (^13^C NMR) nuclear magnetic resonance spectroscopy were determined by analyzing NMR spectra using a Bruker Ascend 500 AVANCE III HD spectrometer. The water-soluble polysaccharide was dissolved in 99% deuterium oxide (D_2_O), and the spectra were recorded at room temperature (^1^H NMR: frequency 500 MHz, acquisition time 3.27 s; ^13^C NMR: frequency 125 MHz, acquisition time 1.1 s). Data are reported in Supplementary Materials.

### 4.4. Radical Scavenging Capacity

#### 4.4.1. DPPH (2,2-diphenyl-1-picrylhydrazyl radical) Assay

The capacity of FUFe to scavenge the free-radical 2,2-diphenyl-1-picrylhydrazyl (DPPH) was determined basically according to the method described by Haddad et al. [6]. One mL aliquots of FUFe at 7 different concentrations (50, 75, 100, 200, 300, 400, and 500 μg/mL) were prepared and mixed to 1 mL of DPPH solution (0.05 g/L in methanol). After a 30 min incubation in darkness, the DPPH radical reduction was evaluated by reading the absorbance at 517 nm using a Gene Quant 1300 UV-VIS spectrophotometer. Ascorbic acid was used as reference standard. The results were calculated as follows: DPPH scavenging activity (%) = [(absorbance of control − absorbance of sample)/(absorbance of control)] × 100. The IC_50_ value, defined as the concentration of FUFe required to cause a 50% decrease in initial DPPH concentration, was estimated by the plot of % of inhibition vs. concentration of FUFe, using a nonlinear regression algorithm (logarithmic) and GraphPad Software (GraphPad Software, Inc., San Diego, CA, USA).

#### 4.4.2. ABTS (2,2-azinobis-3-ethylbenzothiazoline-6-sulfonic acid) Assay

The ABTS assay was performed following the procedure described previously (Benelli et al. 2018), applied to a 96-well microplate. The ABTS^·+^ stock solution was prepared according to Miller and Rice-Evans [45] to obtain a final solution with absorbance about 1 at 734 nm. FUFe at different concentrations (6.25, 12.5, 25, 50, 100, 200, 400 μg/mL) was incubated with ABTS^·+^ for 10 min at room temperature in the dark, then the absorbance was read at 734 nm using a microplate reader (FLUOstar Optima, BMG Labtech microplate reader). Trolox was used as reference and results were expressed as µmol Trolox Equivalents (TE)/g of extract (µmol TE/g). The IC_50_ value, defined as the concentration of FUFe required to cause a 50% reduction in the assay, was estimated by the plot of % of inhibition vs. concentration of FUFe, using a nonlinear regression algorithm (logarithmic) and GraphPad Software.

#### 4.4.3. FRAP (Ferric Reducing Antioxidant Power) Assay

FRAP assay was performed as previously reported [46] by using a 96-well microplate to monitor the reduction of Fe^3+^ tripyridyl triazine (TPTZ) to blue-colored Fe^2+^-TPTZ. Fresh working solution was prepared by mixing 10 volumes of acetate buffer (300 mM, pH 3.6), 1 volume of TPTZ (10 mM in 40 mM HCl), and 1 volume of FeCl_3._6 H_2_O (20 mM). FUFe (concentrations as for ABTS assay) was incubated with FRAP solution for 10 min and the absorbance was read at 593 nm with Trolox as standard as described above.

### 4.5. Cell Culture and Treatments

FaO rat hepatoma cell line (European Collection of Authenticated Cell Cultures, Sigma-Aldrich) was grown in Coon’s modified Ham’s F-12 medium supplemented with L-glutamine and 10% fetal bovine serum (FBS).

Human endothelial cord vein (HECV) cells (Cell Bank and Culture-GMP-IST-Genoa, Italy) were grown in Dulbecco’s modified Eagle’s medium (DMEM) supplemented with L-glutamine and 10% FBS.

Cells were incubated in a humidified atmosphere with 5% CO_2_ at 37 °C. For treatments, cells were grown until 80% confluence, and incubated overnight in serum-free medium with 0.25% bovine serum albumin. To induce intracellular lipid accumulation, cells were treated for 3 h with a mixture of oleate/palmitate (OP) at a final concentration of 0.75 mM (2:1 molar ratio) [18,24]. Thereafter, cells were incubated for 24 h either in control medium (referred to as OP steatotic cells,) or in the presence of FUFe at different concentrations, starting from a FUFe aqueous stock solution (1 mg/mL) diluted with culture medium. Untreated cells were referred as controls.

### 4.6. ROS Production

ROS production in FaO and HECV cells was quantified following the oxidation of the cell-permeant 2′-7′ dichlorofluorescein diacetate (DCF-DA, Fluka, Germany) to 2′-7′dichlorofluorescein (DCF). A stock solution of DCF-DA (10 mM in DMSO) was prepared and stored at −20 °C in the dark. At the end of treatments, cells were scraped and centrifuged (600× *g* for 10 min at 4 °C). After washing with PBS, cells were loaded with 10 μM DCF-DA in PBS and incubated for 30 min at 37 °C in the dark. Then, cells were centrifuged, resuspended in PBS and the fluorescence was measured fluorometrically (λex = 495 nm; λem = 525 nm) in a LS50B fluorimeter (Perkin Elmer, MA, USA) at 25 °C using a water-thermostated cuvette holder. Results were normalized for protein content [47,48].

### 4.7. Quantification of Triglycerides (TGs)

Intracellular TG content was measured in FaO cells using the “Triglycerides liquid” kit (Sentinel, Milan, Italy), as previously described [19,49]. Absorbance was recorded at 546 nm; after chloroform evaporation TG content was determined in which results were recorded spectrophotometrically. For measurement of extracellular TG content, the culture media were processed according to the same method. Values were normalized to protein content and data are expressed as percent TG content relative to controls [50]. For intracellular lipid staining, cells grown on coverslips were rinsed with PBS and fixed with 4% paraformaldehyde for 20 min at room temperature. Neutral lipids were stained by incubation with 1 μg/mL BODIPY 493/503 (Molecular Probes, Life technologies, Monza, Italy) in PBS for 30 min [40]. After washing, nuclei were stained with 4′,6-diamidino-2-phenylindole (DAPI, 5 μg/mL, (ProLong Gold medium with DAPI; Invitrogen). Mounted slides were examined at 10× magnification by Olympus IX53 light microscope (Olympus, Milano, Italy), equipped with the standard epifluorescence filter set up. Representative images were captured with a CCD UC30 camera and a digital image acquisition software (CellSens Entry).

### 4.8. Nitric Oxide (NO) Production

NO production by HECV cells was measured indirectly by spectrophotometric quantification of the end products (nitrites and nitrates, collectively referred as NOx) at 540 nm, using the Griess reaction [51]. NOx accumulation in cell culture media was calculated against a standard curve of sodium nitrite (NaNO_2_) and normalized by protein content (μmol NaNO_2_/mg sample protein) [47].

### 4.9. RNA Extraction and Quantitative Real-Time PCR

Total RNA was extracted by using Trizol Reagent (Sigma-Aldrich, Oakville, ON, Canada, and St. Louis, MO, USA) according to the manufacturer’s instructions. 1 μg of cDNA was synthesized using RevertAid H-Minus M-MuLV Reverse Transcriptase (Fermentas, Hannover, MD, USA) as previously explained [52]. Real-time quantitative (qPCR) reactions were performed in triplicate in a final volume of 25μL using 1× SybrGreen SuperMix and Chromo4TM System apparatus (Biorad, Monza, Italy) as previously described [19]. Primer pairs for the genes under analysis (Table S1) were designed ad hoc and synthesized by TibMolBiol custom oligo synthesis service (Genova, Italy). Amplification conditions were as follows: 3 min at 95 °C, followed by 5 s at 95 °C and 1 min at 60 °C or 64 °C for 40 cycles. A melting curve of qPCR products (65–94 °C) was also performed to ensure the absence of artefacts. The relative quantity of target mRNA was calculated by the comparative Cq method using glyceraldehyde 3-phosphate dehydrogenase (GAPDH) as housekeeping gene and expressed as fold change with respect to controls [53].

### 4.10. Statistical Analysis

Data are means ± S.D. of at least three independent experiments. Statistical analysis was performed using ANOVA with Tukey’s posttest (GraphPad Software, Inc., San Diego, CA, USA).

## 5. Conclusions

In the present work, a new FU purified from the roots of the terrestrial medicinal plant *F. hermonis* has been preliminarily characterized by FTIR and NMR spectra for the first time. FUFe displayed in vitro radical scavenging activity and antioxidant properties in cellular systems such as FaO and HECV cell lines. The antioxidant and antisteatotic actions observed in endothelial and hepatic cells point to candidate FUFe as a possible bioactive compound against NAFLD onset and progression toward more serious conditions that involve vascular damage. *F. hermonis* could be considered as a new extra-marine source for FU purification. Thus, further studies should be planned in order to better define extraction methods, structural characteristics and biological activities, which could also be useful to elucidate the accordance between traditional and current medicine.

## Figures and Tables

**Table 1:** Chemical composition (%) of fucoidan extracted from *F. hermonis*. Data are presented as mean ± S.D. of 3 experiments.

Sulfate	Fucose	Glucose	Galactose	Mannose	Proteins
1.8 ± 1.52	31.5 ± 6.1	29.3 ± 6.32	2.6 ± 5.82	1.2 ± 4.95	0.46 ± 7.6

**Table 2:** Radical scavenging activity (%) of fucoidan extracted from *F. hermonis* (FUFe).

FUFe (µg/mL)	RSA%
50	18.1 ± 4.2
75	26.6 ± 9.2
100	36.4 ± 9.1
200	60.5 ± 11.2
300	72.8 ± 5.1
400	75.7 ± 4.9
500	75.7 ± 4.2
IC_50_ = 157.6 ± 3.3 µg/mL	

RSA = radical scavenging activity (%) = [(absorbance of control − absorbance of sample)/(absorbance of control)] × 100. IC_50_ = The concentration of compound that affords a 50% reduction in the assay. Values represent mean ± S.D. from triplicate experiments.

**Table 3:** Antioxidant activities of FUFe measured by ABTS and FRAP assays.

	ABTS		FRAP
	TEAC (μmol TE/g)	IC_50_ (μg/mL)	TEAC (μmol TE/g)
Fucoidan from *F. hermonis* (FUFe)	410.0 ± 14.0	44.26 ± 2.35	81.30 ± 7.20
Trolox		4.53 ± 1.53	

TEAC = Trolox equivalent (TE) antioxidant concentration. IC_50_ = The concentration of compound that affords a 50% reduction in the assay. Values represent mean ± S.D. from triplicate experiments.

**Table 4:** Maximum RSA of FU extracted from different species at 500 µg/mL.

FU Source	Species	RSA at 500 µg/mL	Reference
Terrestrial plant	*Ferula hermonis*	75.7%	-
	*Eucalyptus globulus*	95.6%	[6]
Brown algae	*Stypopodium schimperi*	44.13%	[14]

RSA = radical scavenging activity (%) = [(absorbance of control − absorbance of sample)/(absorbance of control)] × 100.

## Data Availability

The data presented in this study are available in Supplementary Materials.

## Supplementary Materials

The following are available online, Figure S1: FTIR spectrum of fucoidan isolated from *F. hermonis*. %T: % Transmittance, Figure S2: ^1^H NMR spectrum of fucoidan isolated from *F. hermonis*, Figure S3: ^13^C NMR spectrum of fucoidan isolated from *F. hermonis*, Table S1: Primer pairs used for RT-qPCR analysis.

## Sample Availability

Samples of the compound FUFe are available from the authors.
