# Supplementary material for: Antioxidant and Antisteatotic Activities of a New Fucoidan Extracted from Ferula hermonis Roots Harvested on Lebanese Mountains
Source: Molecules. 2021 Feb 22;26(4):1161. doi: 10.3390/molecules26041161 (PMC7926425; doi:10.3390/molecules26041161)
Supplement: Supplementary file 1 [file molecules-26-01161-s001.pdf]

Supplementary Materials

Antioxidant and antisteatotic activities of a new fucoidan extracted from *Ferula hermonis* roots harvested on Lebanese mountains

Zeinab El Rashed[1,2], Giulio Lupidi[3], Hussein Kanaan[4], Elena Grasselli[1], Laura Canesi[1], Hala Khalifeh[2] and Ilaria Demori[1*]

[1] Department of Earth, Environmental and Life Sciences (DISTAV), University of Genoa, 16132 Genoa, Italy; Zeinab.ALRashed94@hotmail.com (Z.E.R.), elena.grasselli@unige.it (E.G.), laura.canesi@unige.it (L.C.), idemori@unige.it (I.D.)

[2] Rammal Rammal Laboratory (ATAC group), Faculty of Sciences I, Lebanese University, 1003 Beirut, Lebanon.; Zeinab.ALRashed94@hotmail.com (Z.E.R.), hala-khalifeh@hotmail.com (Ha.Kh)

[3] School of Pharmacy, University of Camerino, 62032 Camerino, Italy; giulio.lupidi@unicam.it (G.L.)

[4] Laboratory of Chemical Synthesis and extraction of Polysaccharides from seaweed, Faculty of Pharmacy, Lebanese University, 1003 Beirut, Lebanon; hkanaan@ul.edu.lb (Hu.Ka)

* Correspondence: idemori@unige.it; Tel.: +390103538246

FTIR spectroscopic analysis

Infrared spectroscopy (FTIR) of FUFe was recorded on a Perkin-Elmer FTIR spectrometer Spectrum Two UAT. Data were collected in the range of 4000-400 cm^{-1}.

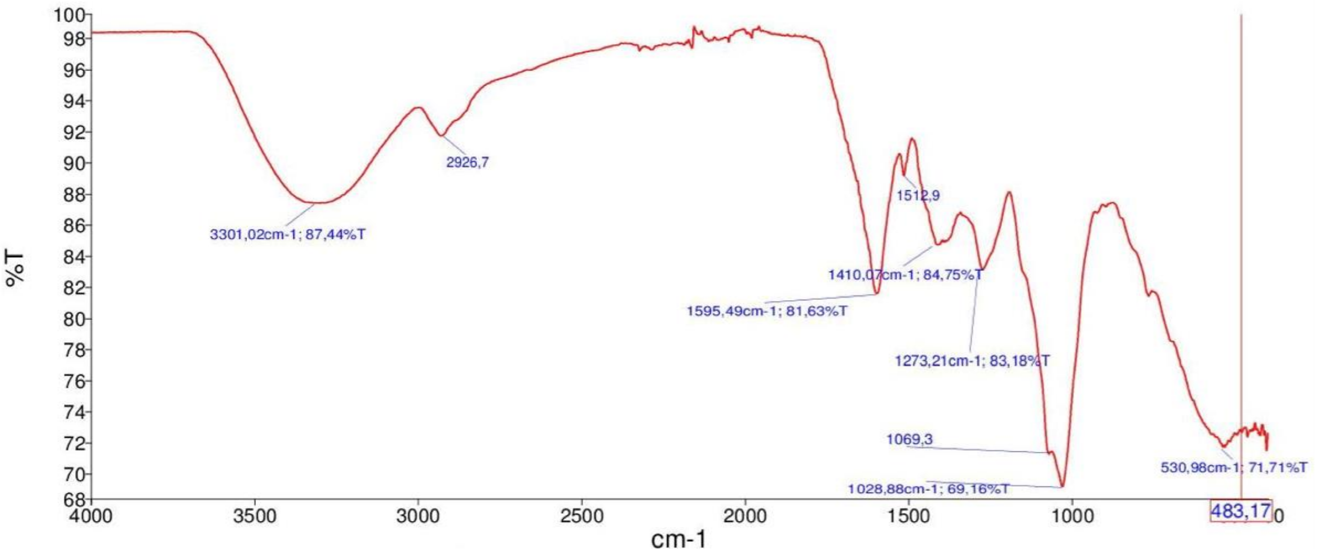

Figure S1. FTIR spectrum of fucoidan isolated from *F. hermonis*. %T: % Transmittance. Fourier Transform

Nuclear magnetic resonance spectroscopy

Proton (^{1}H NMR) and carbon (^{13}C NMR) nuclear magnetic resonance spectroscopy were determined by analyzing NMR spectra using a Bruker Ascend 500 AVANCE III HD spectrometer. The water-soluble polysaccharide was dissolved in 99% deuterium oxide (D_2O), and the spectra were recorded at room temperature (^{1}H NMR: frequency 500 MHz, acquisition time 3.27 sec; ^{13}C NMR: frequency 125 MHz, acquisition time 1.1 sec).

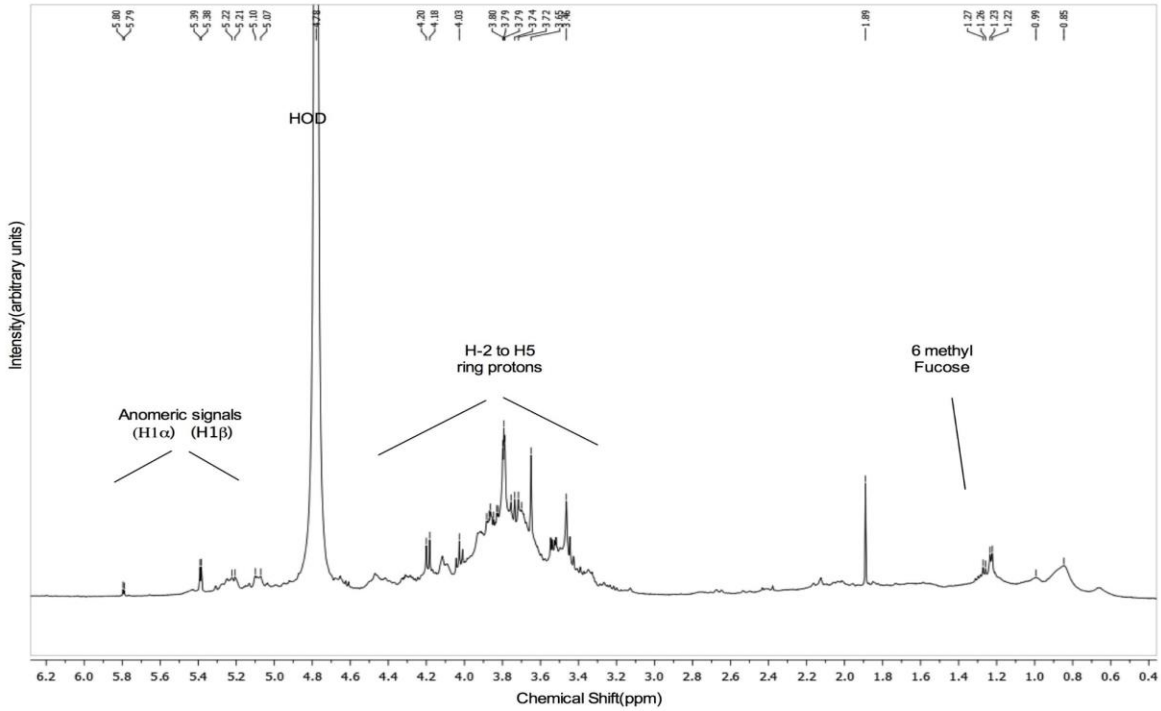

Figure S2: ^{1}H NMR spectrum of fucoidan isolated from *F. hermonis.*

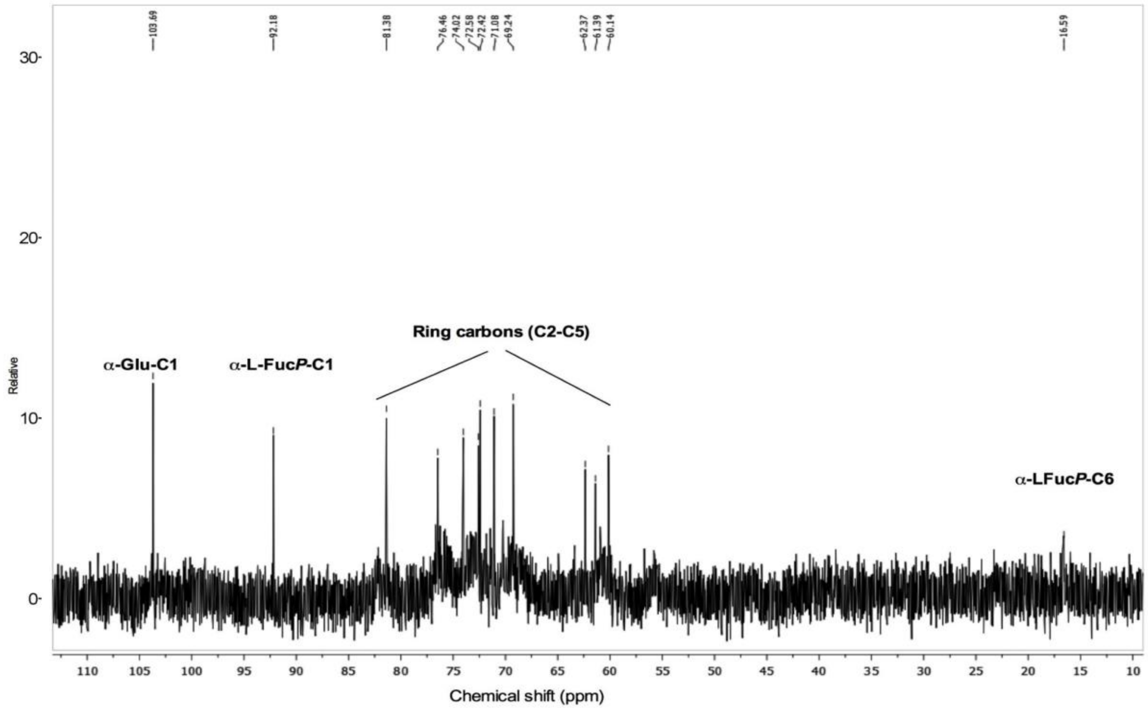

Figure S3. ^{13}C NMR spectrum of fucoidan isolated from *F. hermonis*.

Quantitative real-time PCR

Table S1. Primer pairs used for RT-qPCR analysis

PRIMER NAME	Primer sequence (5′→3′)	Annealing T (°C)	Product lenght (bp)	Accession ID	Ref
GAPDH Fwd	GACCCCTTCATTGACCTCAAC	60	136	DQ403053	[54]
GAPDH Rev	CGCTCCTGGAAGATGGTGATGGG				
PPARα Fwd	CCCCACTTGAAGCAGATGACC	60	139	NM_013196	[54]
PPARα Rev	CCCTAAGTACTGGTAGTCCGC				
PPARγ Fwd	CGGAGTCCTCCCAGCTGTTCGCC	60	116	Y12882	[54]
PPARγ Rev	GGCTCATATCTGTCTCCGTCTTC				
PLIN2 Fwd	CCGAGCGTGGTGACGAGGG	60	148	AAH85861	[55]
PLIN2 Rev	GAGGTCACGGTCCTCACTCCC				
PLIN5 Fwd	GGATGTCCGGTGATCAGAC	60	96	XM_576698	[55]
PLIN5 Rev	GTGCACGTGGCCCTGACCAG				

References

54. Grasselli, E.; Canesi, L.; Voci, A.; De Matteis, R.; Demori, I.; Fugassa, E.; Vergani, L. Effects of 3,5-diiodo-L-thyronine admin-istration on the liver of high fat diet-fed rats. Exp. Biol. Med. 2008, 233, 549–557, doi:10.3181/0710-RM-266.

55. Grasselli, E.; Voci, A.; Pesce, C.; Canesi, L.; Fugassa, E.; Gallo, G.; Vergani, L. PAT protein mRNA expression in primary rat hepatocytes: Effects of exposure to fatty acids. Int. J. Mol. Med. 2010, 25, 505–512, doi:10.3892/ijmm_00000370.
